# Legume Lectins Inhibit Human Parainfluenza Virus Type 2 Infection by Interfering with the Entr

**DOI:** 10.3390/v4071104

**Published:** 2012-06-29

**Authors:** Jun Uematsu, Aoi Koyama, Sayaka Takano, Yukari Ura, Miho Tanemura, Sahoko Kihira, Hidetaka Yamamoto, Mitsuo Kawano, Masato Tsurudome, Myles O’Brien, Hiroshi Komada

**Affiliations:** 1 Microbiology and Immunology Section, Department of Clinical Nutrition, Graduate School of Suzuka University of Medical Science, 1001-1, Kishioka, Suzuka, Mie, 510-0293, Japan; Email: uematsu@suzuka-u.ac.jp; 2 Department of Clinical Nutrition, Faculty of Health Science, Suzuka University of Medical Science, 1001-1 Kishioka, Suzuka, Mie, 510-0293, Japan; Email: komada@suzuka-u.ac.jp (A.K.; S.T.; Y.U.; M.T.); kihira@suzuka-u.ac.jp (S.K.); 3 Faculty of Pharmaceutical Science, Suzuka University of Medical Science, 3500-3, Minamitamagaki, Suzuka, Mie, 513-8670, Japan; Email: ya-moto@suzuka-u.ac.jp; 4 Department of Microbiology, Mie University Graduate School of Medicine, 2-174, Edobashi, Tsu, Mie, 514-8507, Japan; Email: kawanom@doc.medic.mie-u.ac.jp (M.K.); turudome@doc.medic.mie-u.ac.jp (M.T.); 5 Graduate School of Mie Prefectural College of Nursing, 1-1-1 Yumegaoka, Tsu, Mie, 514-0116, Japan; Email: myles.obrien@mcn.ac.jp

**Keywords:** human parainfluenza virus type 2, concanavalin A, lens culinarisis agglutinin, peanut agglutinin, a recombinant green fluorescence protein expressing hPIV-2 without matrix protein

## Abstract

Three lectins with different sugar binding specificities were investigated for anti-viral activity against human parainfluenza virus type 2 (hPIV-2). The lectins, concanavalin A (Con A), lens culinaris agglutinin (LCA) and peanut agglutinin (PNA), inhibited cell fusion and hemadsorption induced by hPIV-2. Virus nucleoprotein (NP) gene synthesis was largely inhibited, but fusion (F) and hemagglutinin-neuraminidase (HN) gene syntheses were not. An indirect immunofluorescence study showed that Con A inhibited virus NP, F and HN protein syntheses, but LCA did not completely inhibit them, and that PNA inhibited only NP protein synthesis. Using a recombinant green fluorescence protein-expressing hPIV-2, without matrix protein (rghPIV-2ΔM), it was found that virus entry into the cells was not completely prevented. The lectins considerably reduced the number of viruses released compared with that of virus infected cells. The lectins bound to cell surface within 10 min, and many aggregates were observed at 30 min. Con A and LCA slightly disrupted actin microfilaments and microtubules, but PNA had almost no effect on them. These results indicated that the inhibitory effects of the lectins were caused mainly by the considerable prevention of virus adsorption to the cells by the lectin binding to their receptors.

## 1. Introduction

Human parainfluenza virus type 2 (hPIV-2) is one of the major human respiratory tract pathogens of infants and children. hPIV-2 is a member of the genus *Rubulavirus* in the family *Paramyxoviridae*, and it possesses a single-stranded non-segmented and negative stranded RNA genome of 15,654 nucleotides [1]. hPIV-2 has 7 structural proteins, nucleoprotein (NP), V, phosphoprotein (P), matrix (M), fusion (F), hemagglutinin-neuraminidase (HN) and large (L) proteins. The gene order of hPIV-2 is 3’-(leader)-NP-V/P-M-F-HN-L-(trailer)-5’. All genes of hPIV-2 were sequenced by our group [2,3,4,5,6,7]. Monoclonal antibodies (mAbs) were made by Tsurudome, and antigenic diversity of clinical isolates was investigated [8]. The infectious hPIV-2 from cDNA clone was constructed by Kawano, and it was shown that its growth property was the same as that of hPIV-2 [9].

Cytoskeleton was reported to have an important role in paramyxovirus replication. Actin microfilaments are important in the hPIV-3 life cycle, specifically at the level of viral transport and replication [10]. Tubulin also acts as a positive transcription factor for *in vitro* RNA synthesis by the Sendai virus [11]. However, the effect of lectins on the cytoskeleton was not reported.

Legume lectins are a family of carbohydrate-binding proteins, and are used as a model system for the study of protein-sugar interaction. Legume lectins can be divided into five groups according to the specificity of the sugar-binding site: fucose (fuc)-specific, *N*-acetyl glucosamine/glucosamine (GlcNAc/Glc) specific, Glucose/mannose (Glc/Man) specific, Galactose/*N*-acetylgalactosamine (Gal/GalNAc) specific, and those that do not bind to any mono-saccharides [12]. Plant lectins have been shown to inhibit the infection of human immunodeficiency virus (HIV) [13,14,15] and cytomegalovirus [13,14]. Plant lectins were reported to inhibit HIV by the prevention of virus adsorption to the cells [16]. However, they also prevent fusion of HIV with their target cells [13,14]. Against severe acute respiratory syndrome (SARS) coronavirus, the mannose binding lectins are the most effective [17]. Recently, Change *et al.* reported that recombinant chimeric lectins consisting of mannose-binding lectins and L-ficolin are potent inhibitors of influenza A virus [18].

In the present study, concanavalin A (Con A) which binds to *N*-linked glycan core trimannoside Mana(-1-6(Man1-3))Man [12,19], lens culinarisis agglutinin (LCA) which binds to core-fucosylated bianntenary *N*-glycans [12,20], and peanut agglutinin (PNA) which binds to Gal-β (1-3)-GalNAc [12] were tested for their ability of virus growth inhibition. Virus RNA was prepared and amplified by polymerase chain reaction (PCR). Virus protein expression was observed by indirect immunofluorescence study using mAbs against NP, F and HN proteins of hPIV-2 [8]. The inhibitory effect of lectins on hPIV-2 entry into the cells and replication in the cells was analyzed using a recombinant green fluorescence protein-expressing hPIV-2 without matrix (M) protein (rghPIV-2ΔM) [9]. The number of viruses released from infected cells cultured with the lectins was determined. The binding of lectins to the cells was observed using rhodamine-labeled lectins. The effects of the lectins on actin microfilaments and microtubules were analyzed using rhodamine phalloidin and anti-tubulin α mAb, respectively.

## 2. Results and Discussion

### 2.1. Inhibitory Effect of the Three Lectins

**Figure 1:**
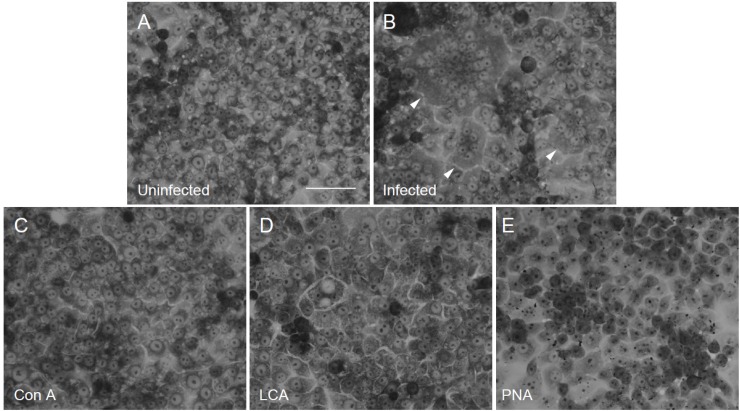
The cell fusion induced by hPIV-2 at four days post infection, and the effect of the three lectins on the cell fusion. Uninfected cells (**A**), hPIV-2 infected cells (**B**) (arrowheads indicate multinucleated giant cells), infected cells cultured with Con A (**C**), LCA (**D**) and PNA (**E**). The three lectins completely inhibited the cell fusion induced by hPIV-2, (bar: 50 μm).

The three lectins were added to the cells and they were infected with hPIV-2, and cell fusion was observed at four days post infection. Figure 1A shows uninfected cells, and Figure 1B multinucleated giant cells induced by hPIV-2. Con A (5 μg/mL) (Figure 1C), LCA (20 μg/mL) (Figure 1D) and PNA (20 μg/mL) (Figure 1E) completely inhibited cell fusion. Also, hemadsorption (Had) was not observed in the lectin-treated hPIV-2-infected cells (data not shown). Rhodamine labeled-Con A (2.5 μg/mL), rhodamine labeled-LCA (20 μg/mL) and rhodamine labeled-PNA (50 μg/mL) completely inhibited both cell fusion and Had (data not shown). The three lectins and three rhodamine labeled-lectins did not disturb normal cell morphology at the concentration used in the experiments.

### 2.2. Viral RNA Synthesis

**Figure 2:**
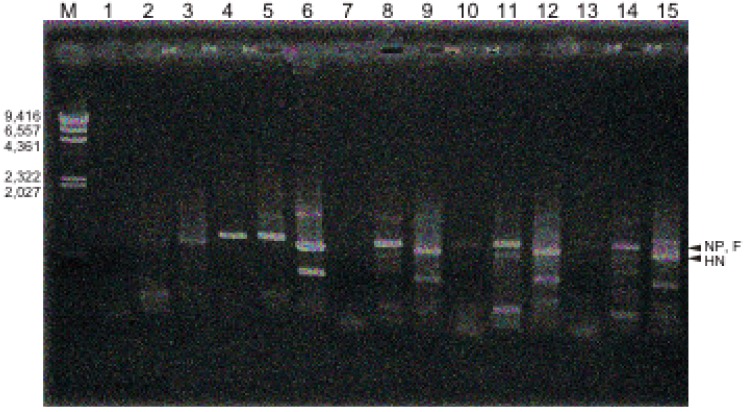
Effect of the three lectins on virus RNA synthesis. Lane M: marker (base pair), lanes 1, 2, 3: uninfected cells, lanes 4, 5, 6: hPIV-2 infected cells, lanes 7, 8, 9: hPIV-2 infected cells cultured with Con A, lanes 10, 11, 12: hPIV-2 infected cells cultured with LCA, and lanes 13, 14, 15: hPIV-2 infected cells cultured with PNA. Lanes 1, 4, 7, 10, 13: NP gene, lanes 2, 5, 8, 11, 14: F gene, lanes 3, 6, 9, 12, 15: HN gene. The lectins largely inhibited NP gene synthesis, but did not inhibit F and HN gene syntheses. The number of base pairs between forward and reverse primers is about 800. Some non-specific bands are observed.

RNA was prepared from the lectin-treated infected cells at four days post infection, and virus-synthesized RNA was analyzed using hPIV-2 specific primers by polymerase chain reaction (PCR). The number of base pairs between forward and reverse primers of NP, F and HN genes was about 800. There are some non-specific bands, some larger and some smaller than 800, and they were also observed faintly in negative control (Figure 2, lanes 1, 2 and 3), and in positive control (Figure 2, lanes 4, 5 and 6). Unexpectedly, ConA almost completely inhibited the synthesis of the NP gene (Figure 2, lane 7), and LCA (Figure 2, lane 10) and PNA (Figure 2, lane 13) largely inhibited NP gene synthesis. However, the syntheses of F (Figure 2, lane 8) and HN (Figure 2, lane 9) genes of hPIV-2 were not inhibited by Con A. Also, F (Figure 2, lane 11) and HN (Figure 2, lane 12) gene syntheses were not inhibited by LCA. PNA did not inhibit the syntheses of F (Figure 2, lane 14) and HN (Figure 2, lane 15) gene. It is difficult to explain the reason why only NP gene synthesis was largely inhibited by the three lectins according to the present understanding of paramyxovirus genome RNA synthesis [1].

### 2.3. Viral Protein Synthesis

**Figure 3:**
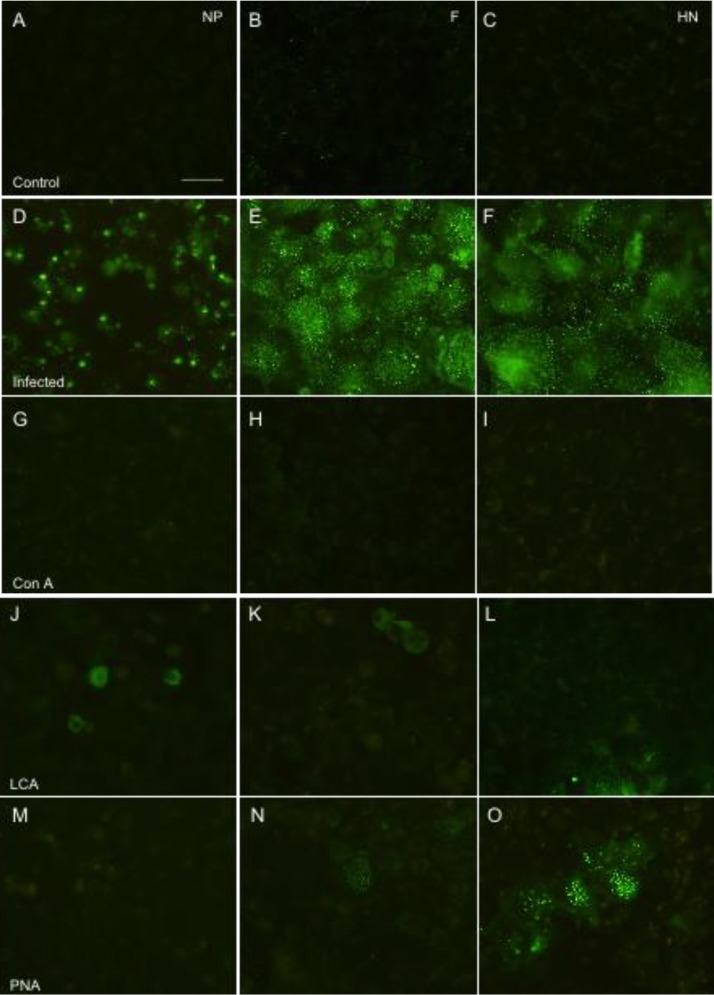
Effect of lectins on the expression of NP, F and HN proteins of hPIV-2. (**A)**, (**B)** and (**C)** are uninfected cells stained with anti-NP, F and HN mAbs, respectively. The expression of NP (**D**), F (**E**) and HN (**F**) proteins of hPIV-2 infected cells: Con A inhibited the expression of NP (**G**), F (**H**) and HN (**I**) proteins. LCA largely inhibited the expression of NP (**J**), F (**K**) and HN (**L**) proteins. PNA inhibited the expression of NP (**M**), largely inhibited F (**N**) protein expression, and only partly inhibited HN (**O**) protein expression, (bar: 50 μm).

Indirect immunofluorescence study was performed to investigate the effect of the three lectins on hPIV-2 protein expression. The lectins were added to the cells and they were infected with hPIV-2. At four days post infection, the cells were fixed and stained with the mAbs against NP, F and HN proteins of hPIV-2. Figure 3A, B and C exhibit uninfected cells stained with the mAbs against NP, F and HN proteins, respectively, and no fluorescence was observed. Figure 3D, E and F show the NP, F and HN protein expression in hPIV-2 infected cells, respectively. In hPIV-2 infected cells, NP, F and HN proteins were observed in almost all the cells: NP protein was observed in many strong fluorescent dots mainly in the cytoplasm, while F and HN proteins were in small dots in the cytoplasm and on the cell surface. Con A inhibited the expression of NP (Figure 3G), F (Figure 3H), and HN (Figure 3I) proteins: NP, F and HN proteins were not observed in the cells. LCA partly inhibited expression of the NP, F and HN proteins (Figure 3J, K and L, respectively). PNA inhibited the expression of NP protein (Figure 3M) and largely inhibited F protein expression (Figure 3N), while HN protein was partly inhibited by PNA (Figure 3O).

As shown in Figure 1, cell fusion was not observed in lectin-treated infected cells. This is because the expressions of F and HN proteins were very little in the cells as observed in Figure 3.

### 2.4. Entry and Replication of hPIV-2

**Figure 4:**
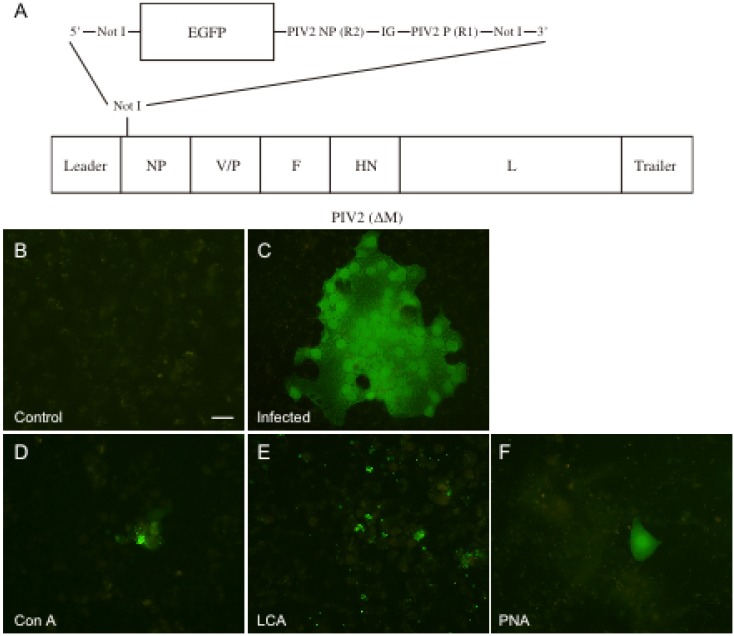
Effect of the lectins on hPIV-2 entry and replication. The organization of the recombinant virus used in this study (**A**). Uninfected cells (**B**), cells infected with rghPIV-2ΔM (**C**), rghPIV-2ΔM infected cells cultured with Con A (**D**), LCA (**E**) and PNA (**F**). The three lectins largely prevented the entry of hPIV-2 into the cells. The small amount of the virus entered into cells, the virus could replicate only in a single cell, but could not infect the neighboring cells (bar: 50 μm).

The above results showed that the three lectins partly inhibited both hPIV-2 RNA and largely inhibited protein syntheses. In the following experiment, we determined the effect of the lectins on the entry and the replication of hPIV-2 using rghPIV-2ΔM (Figure 4). The lectins were added to the cell culture, and the cells were infected with rghPIV-2ΔM (1 × 10^5^ TCID_50_: multiplicity of infection 0.2) and cultured for four days. They were then fixed and observed under a fluorescence microscope. Figure 4A is the organization of rghPIV-2ΔM. Figure 4B is an uninfected negative control. Figure 4C is a positive control: there are many multinucleated giant cells which have strong fluorescence, indicating that rghPIV-2ΔM infected the cells, replicated in the cells and caused cell-to-cell infection. Figure 4D, E and F show the infected cells cultured with Con A, LCA and PNA, respectively. As shown in Figure 4, in several parts of the specimen of the infected cells cultured with the lectins, some positive cells were seen, and around them, many uninfected cells were observed. The results indicate that the lectins considerably prevented virus entry, the small amount of virus entered into cells, the virus could replicate only in a single cell, and could not infect the neighboring cells.

### 2.5. Titration of Virus Released from the Infected Cells

The titers of virus released from the cells cultured with and without the lectins at four days post infection were determined. Without the lectins, the virus titer was 2 × 10^6 ^TCID_50_/mL, and with the lectins it reduced to about 1x10^2 ^TCID_50_/mL, indicating that the lectins largely suppressed the yield of the virus. There were no differences in the effects of the three lectins on the virus yield.

### 2.6. Binding of the Three Lectins to the Cells at the Early Phase of Infection

**Figure 5:**
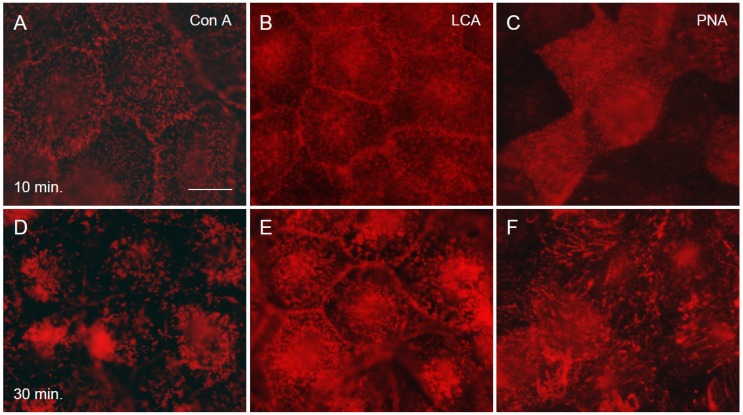
Binding of the three lectins to the cells at the early stage of infection. The three lectins were added to the cells and they were infected with hPIV-2, and cultured for 30 min. Con A (**A**), LCA (**B**) and PNA (**C**) bound to the cell surface within 10 min of addition, and some aggregates were seen at 30 min (Con A: **D**, LCA: **E** and PNA: **F**), (bar: 50 μm).

The binding of the lectins to the cells was analyzed using rhodamine-labeled lectins. The labeled lectins were added to the cells, which were then infected with hPIV-2 and cultured for 30 min. As shown in Figure 5, the lectins bound to the cell surface within 10 min of addition (Figure 5A, B and C for Con A, LCA and PNA, respectively), and some aggregates were observed at 30 min (Figure 5D, E and F for Con A, LCA and PNA, respectively). These results indicated that the lectins bound to the cell surface receptors, and largely prevented the binding of hPIV-2 to the cells non-specifically.

### 2.7. The Effect of the Three Lectins on Cytoskeleton

**Figure 6:**
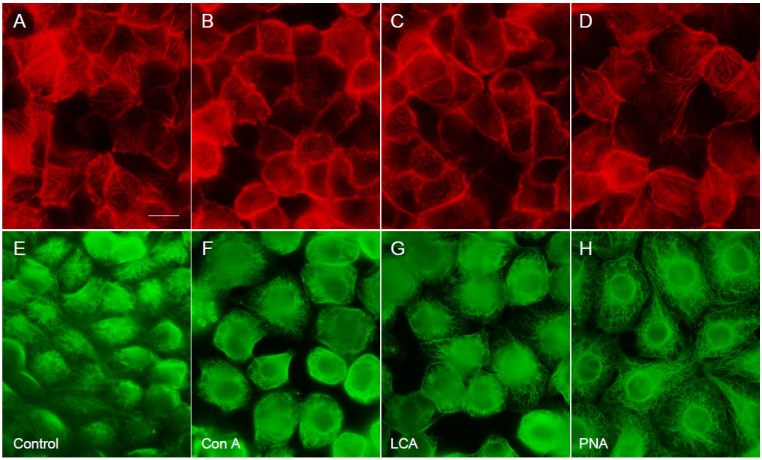
The effect of the three lectins on actin microfilaments and microtubules. The cells were cultured with the lectin for 20 h, and actin microfilaments and microtubules were stained with rhodamine phalloidin (**A-D**) and the anti-tubulin α mAb (**E-H**), respectively. Con A and LCA slightly disrupted both actin microfilaments and microtubules, but PNA did not cause the disruption. A and E show, respectively, actin microfilaments and microtubules of the control cells. B and F show actin microfilaments and microtubules of cells cultured with ConA, C and G are those of the cells cultured with LCA, and D and H those of the cells with PNA, (bar: 50 μm).

The three lectins were added to the cell culture, and the cytoskeleton was observed under an immunofluorescence microscope at 20 h of cultivation. Figure 6A and E show actin microfilaments and microtubules, respectively, in LLCMK_2_ cells. As shown in Figure 6, Con A (Figure 6B for actin and 6F for microtubules) and LCA (Figure 6C for actin and 6G for microtubules) disrupted slightly both actin microfilaments and microtubules. However, PNA exhibited no effect on the cytoskeleton (Figure 6D for actin and 6H for microtubules).

Plant lectins are known to bind to the cell surface sugar moieties, and inhibit virus adsorption to the cells [17]. Mannose- and *N*-acetylglucosamine-specific agglutinins on HIV replication have been reported [13,14]. In addition, it was reported that these lectins were effective on cytomegalovirus [13,14] and that recombinant chimeric lectins inhibited influenza A virus [18]. These plant lectins inhibited virus replication by preventing virus adsorption to the cells. For SARS-coronavirus, the mannose-specific lectins are highly effective, but the inhibitory mechanism is different from that for other viruses. SARS-coronavirus has high-mannose type glycans and the mannose-specific plant lectins can bind to the glycans and prevent the attachment of the virus to the cells [17]. In the present investigation, we analyzed the effect of three legume lectins that have different sugar-binding specificities on the growth of hPIV-2, whose receptor consists of sialic acids [1]. The three lectins bind to each cell receptor, but not to the sialic acids which are the receptor for hPIV-2, so the prevention of hPIV-2 adsorption to the cells must be non-specific. The lectins that bound to the sugar moieties of cell surface might prevent the binding of hPIV-2 by steric hindrance, not by competition. The cytoskeletons were important for virus entry and replication in cells. Actin is necessary for murine leukemia virus entry into cells [21] and for transcription/replication of measles virus [22] and hPIV-3 [10]. Tubulin is also a factor necessary for the synthesis of Sendai virus and vesicular stomatitis virus RNAs [11]. In the present experiment, Con A and LCA induced a slight morphology change in both actin microfilaments and microtubules, indicating that these may be one possibility of reduction of the numbers of the virus released from the infected cells. Taken together, the lectins mainly act on virus entry by considerable prevention of virus binding to the cells.

## 3. Experimental Section

*Lectins:* Con A, LCA and PNA, and rhodamine labeled-Con A, -LCA and -PNA were purchased from Funakoshi (Tokyo, Japan). They were dissolved at 1 mg/mL in 10 mM phosphate buffered saline, pH 7.2 (PBS), and sterilized by filtration.

*Viruses, cell line* *and cultivation of cells:* Virus and recombinant virus were approved by Biosafety committees of Suzuka University of Medical Science. hPIV-2 (Toshiba strain) was used. rghPIV-2ΔM was constructed according to the method described previously [9], and it was shown that rghPIV-2ΔM did not produce infectious virus particles into the culture medium without addition of M protein gene *in trans* (data not shown). The recombinant virus induced multinucleated giant cells with strong fluorescence at four days post infection, however, no progeny virus was found in the medium (data not shown). Titration of rghPIV-2ΔM could be carried out according to the above-mentioned nature of the recombinant virus. LLCMK_2_ cells (rhesus monkey kidney cell line) were cultured in a flat-bottomed 24-well plate in 1 mL culture medium. Minimum essential medium α (MEMα: Wako, Osaka, Japan), supplemented with 2% fetal calf serum (FCS) and 0.1 mg/mL kanamycin, was used. The cells were cultured at 37 °C in a humidified atmosphere with 5% CO_2_. After three days, when the cells became confluent (5 × 10^5^ cells), the medium was changed to MEMα with 0.5% FCS and 0.1 mg/mL kanamycin. The lectins were added to the cells, and the cells were infected with hPIV-2 (3 × 10^2^ TCID_50_).

*Cytopathogenic assay:* Cell fusion and Had were observed at four days post infection. Cell fusion was observed on the cells stained with 1% methyl blue. A Had test was carried out using sheep red blood cells (SRBC). The cells were incubated with 0.4% SRBC at room temperature for 30 min, washed four times with PBS, and Had was observed under a light microscope for cell culture.

*RNA preparation, cDNA synthesis and PCR:* RNA was extracted from the cells (2 × 10^6^ cells) cultured in a flat-bottomed 6-well plate using TRIZOL reagent (Invitrogen, CA, USA) according to the manufacturer’s method. cDNA was synthesized with 2 μg of RNA using superscript II reverse transcriptase (Invitrogen), with forward primers for NP, F and HN genes of hPIV-2 [23]. PCR was carried out with cDNA using forward and reverse primers for NP, F and HN genes [23] and Ex Taq (Takara, Shiga, Japan).

*Immunofluorescence study:* To detect virus proteins in the infected cells, the cells were fixed with 10% formaldehyde-PBS at room temperature for 15 min, washed with PBS, and incubated with mouse mAbs against NP, F and HN proteins of hPIV-2 [8] at room temperature for 30 min. After washing with PBS, the cells were incubated with Alexa 488 conjugated secondary antibody to mouse IgGs (Invitrogen) at room temperature for 30 min, and observed under a fluorescence microscope (Olympus, Tokyo, Japan).

To determine the binding of lectins to the cells at the early phase of infection, rhodamine labeled lectins were added, the cells were infected with hPIV-2 and cultured for 30 min. They were fixed at 10 and 30 min after infection.

Actin was detected by using rhodamine phalloidin (Invitrogen), and microtubule by using anti-tubulin α mAb against sea urchin tubulin α (clone B-5-1-2, Sigma-Aldrich, St Louis, MO, USA).

## 4. Conclusion

The effects of lectins on hPIV-2 infection were analyzed, and we showed that the lectins had a partial inhibitory effect on virus RNA synthesis and they largely inhibited protein syntheses. In addition, it was shown that the lectins non-specifically prevented the entry of hPIV-2 into cells by binding to the cell surface lectin receptors.
